# Zero Time‐to‐Echo Imaging of the Hand and Wrist in Rheumatoid Arthritis Patients

**DOI:** 10.1002/jmri.70420

**Published:** 2026-07-09

**Authors:** Jun Tsukamoto, Akitaka Fujisaki, Yoshiko Hayashida, Seiichi Murakami, Atsushi Nagayasu, Yasuyuki Todoroki, Shingo Nakayamada, Yoshiya Tanaka, Takatoshi Aoki

**Affiliations:** ^1^ Department of Radiology University of Occupational and Environmental Health Kitakyushu Fukuoka Japan; ^2^ Department of Radiological Science, Faculty of Health Sciences Junshin Gakuen University Fukuoka Japan; ^3^ The First Department of Internal Medicine University of Occupational and Environmental Health Kitakyushu Fukuoka Japan

**Keywords:** computed tomography, CT‐like image, magnetic resonance imaging, rheumatoid arthritis

## Abstract

**Background:**

Zero time‐to‐echo (ZTE) imaging allows for signal acquisition from tissues with extremely short T2* relaxation times, enabling the generation of computed tomography (CT)‐like images through gray‐scale inversion.

**Purpose:**

To assess the usefulness of adding ZTE imaging to conventional MRI for the detection of hand and wrist bone erosions in rheumatoid arthritis (RA) patients, using CT as the reference method.

**Study Type:**

Prospective.

**Subjects:**

Fifteen patients with RA (4 male and 11 female; mean age, 61.8 ± 15.5 years) participated in this study.

**Field Strength/Sequence:**

3‐T, T1‐weighted image (fast spin echo), T2‐weighted image (fast spin echo), ZTE imaging (gradient echo).

**Assessment:**

Three radiologists independently evaluated bone erosions on plain radiography, conventional MRI, and ZTE imaging using a four‐point scale (0 = normal; 1 = discrete erosion; 2 ≤ 50% of joint surface involvement; 3 ≥ 50%). Bone erosion was defined as a sharply delineated juxta‐articular lesion with a cortical defect. A total of 25 anatomical sites per hand and wrist were assessed, including the distal radius and ulna, eight carpal bones, metacarpal bases, and proximal and distal aspects of the metacarpophalangeal joints. CT was interpreted by a musculoskeletal radiologist and served as the reference standard.

**Statistical Tests:**

Diagnostic performance (sensitivity and specificity) was compared with the McNemar test, and interobserver variability was assessed using Fleiss κ statistics. *p* values less than 0.01 were considered significant.

**Results:**

The sensitivity and specificity of plain radiography, conventional MRI, and ZTE were 40.6/98.9%, 72.6/94.6%, and 95.9/97.6%, respectively. Interobserver agreement on bone erosion assessment was excellent for ZTE imaging (*κ* value of 0.822) but fair for plain radiography (*κ* value of 0.427) and good for conventional MRI (*κ* value of 0.711).

**Data Conclusions:**

ZTE imaging can provide reliable bone erosion assessment in RA patients.

**Level of Evidence:**

2.

**Technical Efficacy:**

Stage 2.

## Plain Language Summary

Accurate detection of bone erosions is important in rheumatoid arthritis (RA). In this prospective study, adding zero time‐to‐echo (ZTE) imaging significantly improved the detection of bone erosions compared with conventional MRI. When CT was used as the reference standard, ZTE demonstrated high sensitivity, specificity, and overall accuracy. ZTE also improved agreement between radiologists. These findings suggest that ZTE imaging may serve as a reliable and radiation‐free alternative to CT for evaluating bone erosions in patients with RA.

## 1 Introduction

Rheumatoid arthritis (RA) is a chronic inflammatory disease that causes synovial joint damage, disability, and reduced life expectancy [1]. Recognition of RA as early as possible is important because a considerable proportion of patients develop irreversible joint damage soon after disease onset [2]. The presence of bone erosions suggests highly active RA, and the progression of the erosive process after treatment necessitates therapeutic changes [3, 4]. Thus, assessment for bone erosions is important for both the early diagnosis and the management of RA patients. Plain radiography of the hand and wrists has traditionally been used for the clinical assessment of RA patients. According to the international recommendations of the American College of Rheumatology (ACR) [5] and the European League Against Rheumatism (EULAR) [6], plain radiography remains the reference method for the diagnosis and follow‐up of RA patients. However, overlapping bony structures make some regions difficult to evaluate, and plain radiography has limited sensitivity for detecting bone erosions [7, 8].

MRI has been increasingly recognized as a useful method for diagnosis and monitoring of therapeutic response in RA patients since it enables clinicians to visually detect bone erosion and active synovitis in early RA before the changes are visible on plain radiographs [3]. On the other hand, several researchers have reported that conventional MRI has lower sensitivity to bone erosion than computed tomography (CT) [7, 9, 10, 11]. A previous study has reported that MRI has reduced sensitivity for detecting bone erosions in the carpal bones, particularly in the pisiform, trapezium, and hamate, when compared with CT [9]. Moreover, MRI has been shown to miss approximately 9% of erosions detected by CT at the metacarpal bases [10].

Recently, zero time‐to‐echo (ZTE) imaging has been developed to detect such low signals from tissues with extremely short T2* values. After gray‐scale inversion, this sequence can generate CT‐like images from MRI [12]. Although the use of ZTE is expected to overcome the weakness of MRI in bone evaluation, to our best knowledge, no studies have been conducted on the use of ZTE images for evaluating bone erosion in RA patients.

The purpose of this study is to prospectively evaluate the usefulness of adding ZTE imaging to conventional MRI for detecting hand and wrist bone erosions in RA patients, using CT as the reference standard.

## 2 Material and Methods

This study was approved by our institutional review board (number 22–090). Written informed consent was obtained from all participants.

### 2.1 Patients

From December 2022 to October 2023, 15 consecutive patients with RA fulfilling the RA classification criteria by the American College of Rheumatology criteria/European League Against Rheumatism (EULAR) [13] were prospectively enrolled in this study. The patients met all of the following inclusion criteria: (a) presentation with polyarthralgia including wrist joint, (b) underwent plain radiography, CT examination, conventional MRI examinations, and ZTE sequences of the symptomatic hand and wrist within 3 weeks, and (c) fulfillment of the ACR diagnostic criteria as conclusively determined by certificated rheumatologists.

### 2.2 Image Data Acquisition

#### 2.2.1 Plain Radiography

The digital radiographic examination (Flat panel detector, KONICA MINOLTA), including posteroanterior and oblique views of the bilateral hand and wrist, was obtained at a tube voltage of 50 kVp and a tube current of 200 mA, according to the standard protocol of our department. The mean total radiation dose was 0.04 mGy per patient.

#### 2.2.2 CT Examination

Bilateral wrist and hand CT scan was obtained by using a 320‐row multi‐slice CT device (Aquilion ONE; Canon Medical Systems, Tochigi, Japan), and an 80‐row multi‐slice CT device (Aquilion PRIME; Canon Medical Systems, Tochigi, Japan). The patient was positioned prone with the dominant arm outstretched, palm facing downward. All exposures were standardized according to the standard protocol used in our department (120 kV on average, X‐ray tube rotation speed of 1.0 s, beamwidth of 320 × 0.5 mm, volume CT dose index of 16.4–18.4 mGy, CT‐Automatic Exposure Control [AEC] setting target SD of 9.5–10 Hounsfield units for a 1‐mm image of the hand standard kernel FC14). Axial sections 1 mm thick were obtained, and these were used to reconstruct sagittal, coronal, and axial images 1 mm thick. CT scan scoring was performed on 1‐mm sections. The mean dose‐length product was 7.15 mGy · cm (range, 6.5–8.1 mGy cm).

#### 2.2.3 MRI Protocol

MRI was performed with a 3.0‐T MRI (SIGNA Premier, GE Healthcare, Milwaukee, WI, USA) with GEM small flex coil (16‐M Array; NeoCoil, Pewaukee, Wisconsin, USA) in accordance with the outcome measures in rheumatology (OMERACT) scoring system recommendations [14], unenhanced T1‐weighted images, contrast‐enhanced fat‐suppressed T1‐weighted images (2D multipoint DIXON, repetition time/echo time (TR/TE), 683/13 ms; matrix size, 400 × 340; Field of View (FOV), 24 cm) that enable visualization in 2 planes, and fat‐suppressed fast spin‐echo T2‐weighted sequences (2D multipoint DIXON, TR/TE, 5377/81 ms; matrix size, 400 × 352; FOV, 24 cm) that enable visualization in two planes were included. Contrast‐enhanced images were obtained after bolus injection of gadoteridol, meglumine gadoterate, or gadobutrol into a vein in the contralateral arm. Contrast agents were used during MRI acquisition for clinical purposes, but the evaluation of bone erosions in this study was based exclusively on non–contrast‐enhanced sequences.

The 3D ZTE sequence was performed with the following parameters: flip angle, 1°; TR/TE, 529/0 ms; reconstruction matrix, 300 × 300; FOV, 22 cm; and pixel bandwidth, 326 Hz/pixel. The section thickness was 1.5 mm for coronal images and 2.0 mm for axial images, and the total acquisition time was 5 min 49 s, covering both bilateral hands and wrists. Patients were positioned laterally, with both hands held together in a prayer‐like position, and the hands were placed between two coils for signal reception.

### 2.3 Image Evaluation

The MR images and the radiographs were independently interpreted by three certified radiologists (Y.H., A.F., and J.T.) with 28, 13, and 7 years of experience in the imaging evaluation of arthritis, respectively. The three readers (Y.H., A.F., and J.T.) independently and consecutively read plain radiography, conventional MRI (axial and coronal T1‐weighted and fat‐suppressed T2‐weighted images), and ZTE images. The readers were blinded to clinical and imaging modalities (including those performed on the same day and prior images). Images obtained at plain radiography, conventional MRI, and ZTE were separated by imaging modality into three groups of patients, each randomly assigned. The three readers independently and consecutively read the radiographic, conventional MRI, and ZTE images, with a one‐week interval between each imaging modality group. Original ZTE images are primarily used for evaluation, but reformatted ZTE images are available for review if required. The CT scan, regarded as the reference standard, was analyzed by the experienced musculoskeletal radiologist (T.A.), who has 32 years of expertise. The readers were blinded to clinical and imaging modalities (including those performed on the same day and prior images). Bone erosion was considered to be a sharply delineated juxta‐articular lesion with a cortical defect. They analyzed the erosion by using a 4‐point scale of erosion (0, normal; 1, discrete erosion; 2, < 50% of the joint surface; 3, > 50% of the joint surface). Images were evaluated for bone erosions at 25 anatomical sites per finger and wrist, including the distal radius, distal ulna, eight carpal bones, first through fifth metacarpal bases, and the proximal and distal aspects of the first through fifth metacarpophalangeal joints [14]. For comparison of detection rates between modalities, a lesion was considered positive if at least one observer identified an erosion; the other observers then reviewed the site, and any discrepancies were resolved by interobserver agreement. For the CT images, erosions were assessed by using axial, coronal, and sagittal reformations with the same criteria.

### 2.4 Statistical Analysis

Comparison of bone erosion was analyzed statistically with the Kruskal–Wallis test, followed by the non‐parametric Mann–Whitney *U* test for multiple comparisons and Bonferroni correction. Diagnostic performance (sensitivity and specificity) using CT as the reference standard was compared using the McNemar test. Interobserver variability for bone erosions was calculated using Fleiss *κ* statistics, and 95% confidence intervals were obtained for kappa values. The strength of agreement was considered slight for kappa values of 0.400 or less, fair for kappa values of 0.40–0.60, good for kappa values of 0.60–0.80, and excellent for kappa values of 0.81 or greater. All calculations were performed using IBM SPSS Statistics (ver.31.0, IBM, Armonk, NY). *p* values less than 0.01 were considered significant in all analyses.

## 3 Results

### 3.1 Bone Erosion Detection Rate

Eleven female patients (mean age, 56.3 years; median, 61 years; range, 26–78 years), and 4 male patients (mean age, 77 years; median, 77 years; range, 70–84 years), with a mean disease duration of 79.9 months (±49.6 (SD)) participated in this prospective study (Table 1). The detection rate of bone erosion for plain radiography, conventional MRI, and ZTE images was 11.5%, 23.1%, 26.9%, respectively (Table 2). Significantly more bone erosions were revealed using ZTE than with plain radiography or conventional MRI (Figures 1, 2, 3).

**TABLE 1:** Patient characteristics.

Characteristic	(*n* = 15)
Age (years)^a^	61.8 ± 15.5
Male	4
Female	11
Mean disease duration (months)^a^	79.9 ± 49.6
SDAI^a^	28.1 ± 16.8
CDAI^a^	26.8 ± 15.8

Abbreviations: CDAI; Clinical Disease Activity Index; SDAI; Simplified Disease Activity Index.

^a^ Means ± SDs.

**TABLE 2:** Detection rate of bone erosion in rheumatoid arthritis patients.

Radiography	Conventional MRI	ZTE	*p* *	*p* **	*p* ***
11.5 (86/750)	23.1 (173/750)	26.9 (202/750)	< 0.001	< 0.001	< 0.001

*Note:* Except where indicated, data are presented as percentages; numerators and denominators are shown in parentheses.

Abbreviations: MRI = magnetic resonance imaging, ZTE = zero time‐to‐echo.

* *p* value for comparison among radiography and conventional MRI.

** *p* value for comparison among radiography and ZTE.

*** *p* value for comparison among conventional MRI and ZTE.

**FIGURE 1:**
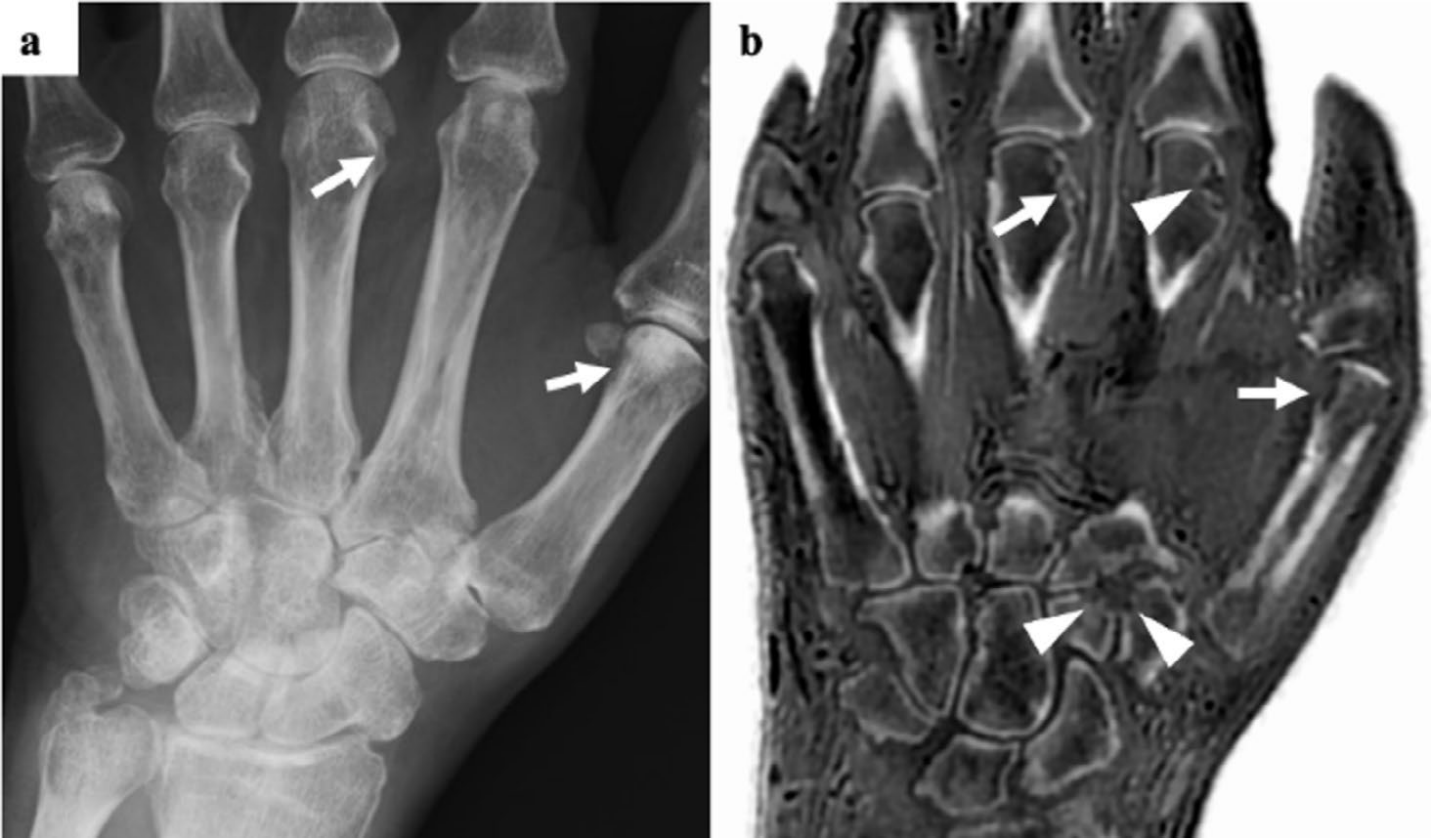
Fifty‐year‐old man with rheumatoid arthritis. Radiography (a) demonstrates bone erosions at the distal ends of the first and third metacarpals, and coronal zero time‐to‐echo (ZTE) image (b) shows more clearly the erosions (arrows). In addition, the other erosions are seen on the distal ends of the second metacarpals and the trapezium and trapezoid bones (arrowheads), which are difficult to detect on radiography.

**FIGURE 2:**
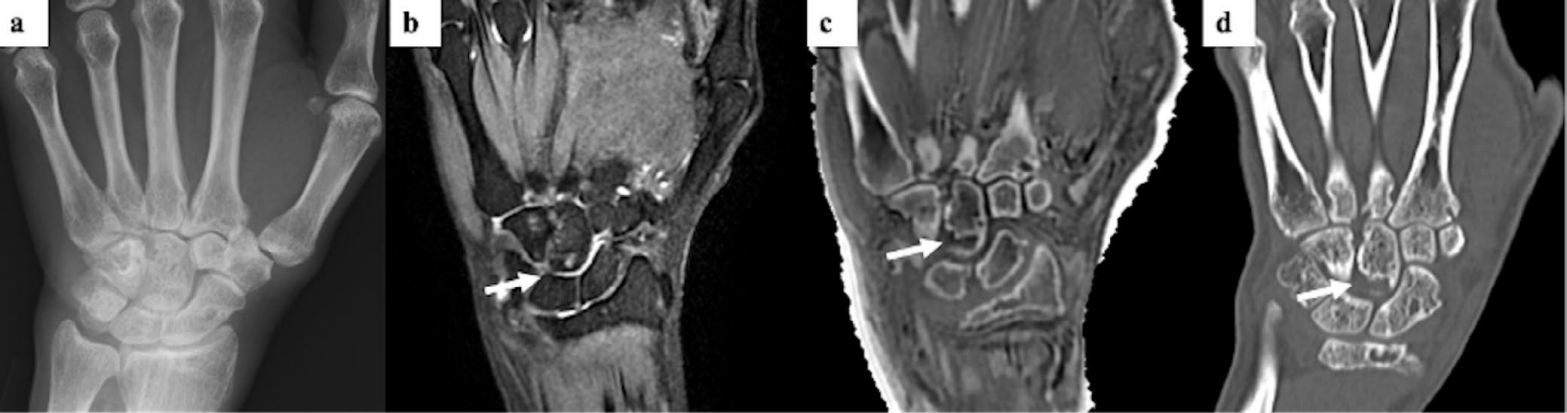
Sixty‐year‐old woman with rheumatoid arthritis. Bone erosion in the capitate bone (arrows) is more clearly delineated on the coronal ZTE image (c) and CT (d) image than on the radiograph (a) and the fat‐suppressed T2‐weighted image (b) (arrows).

**FIGURE 3:**
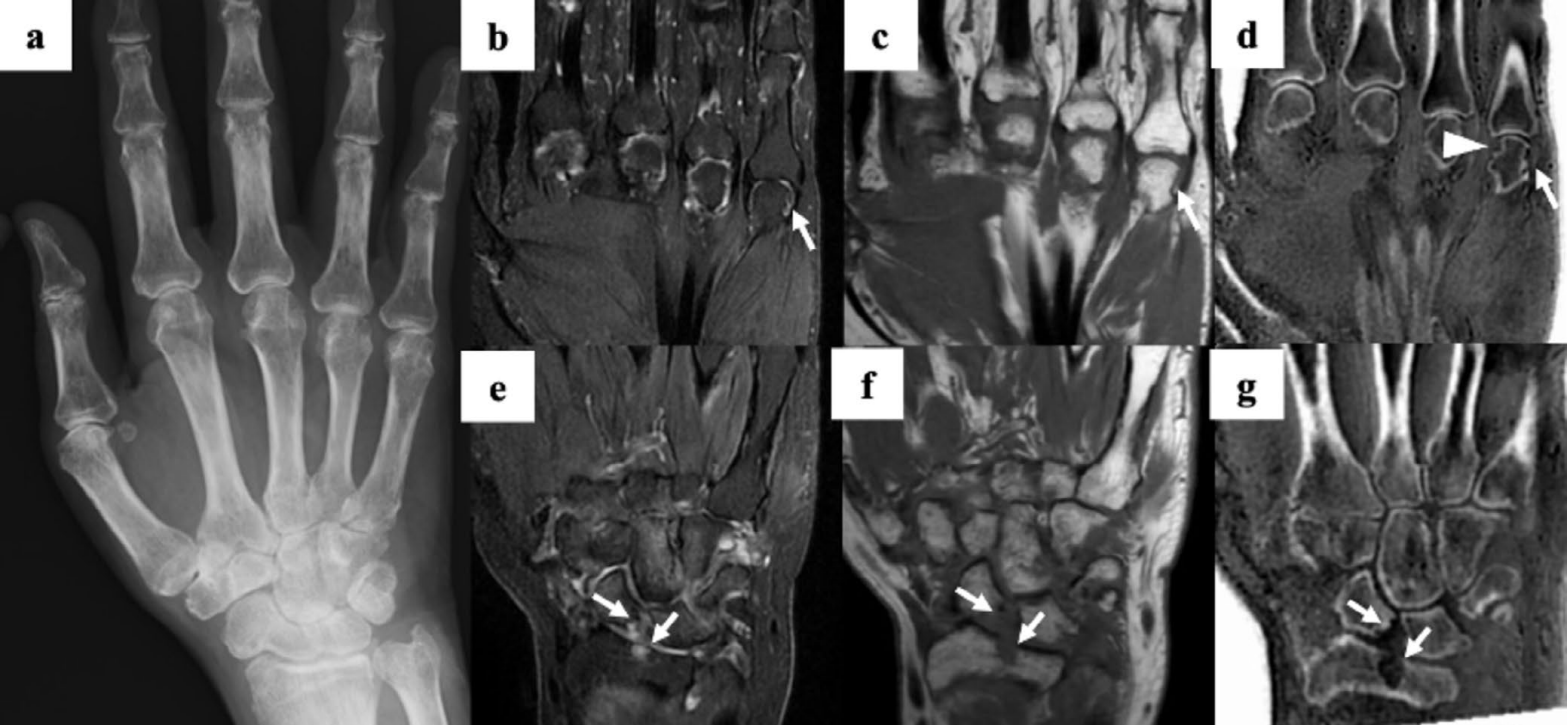
Seventy‐year‐old man with rheumatoid arthritis. Arrows indicate bone erosions not detected on radiography (a), and an arrowhead indicates bone erosions not detected on either radiography or conventional MRI (b, e: fat‐suppressed T2‐weighted coronal image, c, f: T1‐weighted coronal image). The bone erosion at the radial side of the distal aspect of the fifth metacarpal bone (arrowhead) is visualized only on coronal ZTE (d). Other erosions indicated by arrows are also delineated more clearly on ZTE (d, g) than on conventional MRI (b, c, e, f).

### 3.2 Diagnostic Performance Using CT as the Reference Standard

When CT was used as the reference standard, the sensitivity, specificity, and accuracy were 40.6%, 98.9%, and 83.6%, respectively, for plain radiography, 72.6%, 94.6%, and 88.8%, respectively, for conventional MRI, and 95.9%, 97.6%, and 97.2%, respectively, for ZTE. Table 3 summarizes the diagnostic performance using CT as the reference standard. Pairwise comparisons using the McNemar test demonstrated that ZTE had significantly higher sensitivity than conventional MRI.

**TABLE 3:** Diagnostic performance of radiography, conventional MRI and ZTE for bone erosion.

	Radiography	Conventional MRI	ZTE	*p* *	*p* **	*p* ***
Sensitivity	40.6 (80/197)	72.6 (143/197)	95.9 (189/197)	< 0.001	< 0.001	< 0.001
Specificity	98.9 (547/553)	94.6 (523/553)	97.6 (540/553)	< 0.001	0.143	0.009

*Note:* Except where indicated, data are presented as percentages; numerators and denominators are shown in parentheses.

Abbreviations: MRI = magnetic resonance imaging, ZTE = zero time‐to‐echo.

* *p* value for comparison among radiography and conventional MRI.

** *p* value for comparison among radiography and ZTE.

*** *p* value for comparison among conventional MRI and ZTE.

### 3.3 Interobserver Agreement

Interobserver agreement for bone erosions is summarized in Table 4. Interobserver agreement on bone erosion assessment was excellent for ZTE imaging (*κ* = 0.822; 0.788–0.856) but was not excellent for plain radiography (*κ* = 0.427; 0.388–0.466) and conventional MRI (*κ* = 0.711; 0.678–0.745). By site evaluation, at the distal and the proximal MCP joints, interobserver agreement for bone erosion was excellent for ZTE imaging (*κ* = 0.883 and 0.858) but was not excellent for conventional MRI (*κ* = 0.649 and 0.793). ZTE also improved interobserver agreement for the metacarpal bases, the carpal bones, and the distal radius and ulna (*κ* = 0.785, 0.782, and 0.868) compared with conventional MRI (*κ* = 0.656, 0.649, and 0.804).

**TABLE 4:** Interobserver variability for bone erosion.

Sites	Interobserver variability (*κ*)		
	Radiography	Conventional MRI	ZTE
Distal MCP joints	0.383 (95% CI: 0.290–0.475)	0.649 (95% CI: 0.559–0.738)	0.883 (95% CI: 0.792–0.973)
Proximal MCP joints	0.581 (95% CI: 0.495–0.667)	0.793 (95% CI: 0.712–0.873)	0.858 (95% CI: 0.772–0.943)
Metacarpal bases	0.401 (95% CI: 0.315–0.486)	0.656 (95% CI: 0.580–0.731)	0.785 (95% CI: 0.708–0.861)
Carpal bones	0.378 (95% CI: 0.310–0.446)	0.649 (95% CI: 0.591–0.706)	0.782 (95% CI: 0.724–0.839)
Distal radius and ulna	0.309 (95% CI: 0.170–0.448)	0.804 (95% CI: 0.696–0.913)	0.868 (95% CI: 0.759–0.977)
All sites	0.427 (95% CI: 0.388–0.466)	0.711 (95% CI: 0.678–0.745)	0.822 (95% CI: 0.788–0.856)

Abbreviations: MRI = magnetic resonance imaging, ZTE = zero time‐to‐echo.

## 4 Discussion

This prospective study demonstrates the substantial benefits of incorporating ZTE imaging into conventional MRI, enhancing its diagnostic capability compared with plain radiography and conventional MRI alone. In addition, the results indicate that the diagnostic performance of ZTE for detecting bone erosion is comparable to that of CT. The improved sensitivity reduces the likelihood of missing bone erosions, which is particularly important in conditions such as RA where early detection can influence treatment outcomes. Furthermore, the high interobserver agreement observed with ZTE suggests that this technique may facilitate more consistent evaluations among radiologists, potentially leading to standardized diagnostic approaches in clinical practice. We believe that incorporating ZTE into conventional MRI improves its utility for assessing bone erosions in RA patients compared with conventional MRI alone.

Although interobserver agreement for bone erosions was fair with plain radiography and good for conventional MRI, it was excellent with ZTE imaging. According to previous reports, the sensitivity of MRI for detecting bone erosions in the carpal bones, as well as in the metacarpal bases, is lower than that of CT [7, 9]. ZTE imaging improved interobserver agreement even at these sites compared with conventional MRI. The boundaries of bone erosions are clearly delineated on CT‐like images produced by ZTE, which are not affected by osteitis/bone marrow edema, joint effusion, or synovial thickening that can complicate the interpretation of conventional MRI [15, 16]. Moreover, 3D volume data acquisition enables the generation of arbitrary cross‐sections at high resolution, allowing for detailed analysis of complexly shaped wrist joints [17, 18]. These advantages of ZTE may account for the improved interobserver agreement observed in this study.

When assessing bone erosions of the hand and wrist on ZTE images, the main challenge encountered in this study was differentiating true bone erosions from vascular foramina, particularly in anatomically complex regions. It should be recognized that vascular foramina may appear as areas of low signal intensity on ZTE images, mimicking bone erosions. Cross‐checking with other MRI sequences and identifying a linear low‐signal pattern of vessels extending from the extraosseous to the intraosseous regions may help overcome this pitfall.

The consensus among researchers is that CT is the optimal imaging modality for identifying bone erosions in RA patients because of its multiplanar capabilities and improved cortical definition [10, 19]. However, CT is not routinely used in RA patients due to several drawbacks, including cost, radiation exposure, and limited access compared with plain radiography [9, 10, 20]. Three‐dimensional ZTE can evaluate bone erosion at any cross‐section by adding less than 6 min of scanning time to conventional MRI, allowing complete coverage of bilateral hands and wrists. This technique can be readily incorporated into the clinical workflow and enables simultaneous assessment of other MR findings relevant to RA evaluation, such as synovitis and bone marrow edema, within a single examination. ZTE is therefore a promising technique for RA patients, offering a means to avoid unnecessary radiation exposure, reduce costs, and eliminate the need for additional CT examination.

## 5 Limitations

First, this study included a rather small number of cases from a single institution. A larger multicenter study would be expected to provide more comprehensive and generalizable results. Second, although RA is a systemic disease, we did not assess regions other than the hands and wrists because they are among the earliest and most frequently involved sites in RA patients. Third, although contrast material was administered as part of the MRI protocol, contrast‐enhanced images were not used for analysis. Postcontrast images can enhance the detection of synovitis and tenosynovitis in RA patients. However, according to the OMERACT recommendations, the recommended MRI sequences, which are primarily used to assess bone erosions, include non‐contrast‐enhanced T1‐weighted images, and contrast‐enhanced images are not mandatory [21, 22]. Because our study primarily focused on the efficacy of ZTE in detecting bone erosions, we consider the exclusion of postcontrast images to be appropriate. Finally, the prognostic implications of improved bone erosion detectability with ZTE remain unclear. Longitudinal studies investigating the prognostic value of ZTE findings in relation to treatment outcomes will be warranted for determining its clinical relevance.

## 6 Conclusion

The integration of ZTE with conventional MRI enhances the accuracy and reproducibility of bone erosion detection in RA, achieving diagnostic performance comparable to that of CT. The most important issue in the management of RA is the early intervention before bone erosion occurs. Therefore, clarifying bone erosion by ZTE can contribute to the appropriate management of RA patients.

## Funding

The authors have nothing to report.

## Conflicts of Interest

The authors declare no conflicts of interest.

## References

[1] J. S. Smolen , D. Aletaha , and I. B. McInnes , “Rheumatoid Arthritis,” Lancet 388, no. 10055 (2016): 2023–2038. 10.1016/S0140-6736(16)30173-8 27156434

[2] B. Combe , “Progression in Early Rheumatoid Arthritis,” Best Practice & Research. Clinical Rheumatology 23, no. 1 (2009): 59–69. 10.1016/j.berh.2008.11.006 19233046

[3] F. McQueen , M. Lassere , J. Edmonds , et al., “OMERACT Rheumatoid Arthritis Magnetic Resonance Imaging Studies. Summary of OMERACT 6 MR Imaging Module,” Journal of Rheumatology 30, no. 6 (2003): 1387–1392. 12784423

[4] Y. Tanaka , “Managing Osteoporosis and Joint Damage in Patients With Rheumatoid Arthritis: An Overview,” Journal of Clinical Medicine 10, no. 6 (2021): 1241. 10.3390/jcm10061241 PMC8002542 33802804

[5] F. C. Arnett , S. M. Edworthy , D. A. Bloch , et al., “The American Rheumatism Association 1987 Revised Criteria for the Classification of Rheumatoid Arthritis,” Arthritis and Rheumatism 31, no. 3 (1988): 315–324. 10.1002/art.1780310302 3358796

[6] B. Combe , R. Landewe , C. Lukas , et al., “EULAR Recommendations for the Management of Early Arthritis: Report of a Task Force of the European Standing Committee for International Clinical Studies Including Therapeutics (ESCISIT),” Annals of the Rheumatic Diseases 66, no. 1 (2007): 34–45. 10.1136/ard.2005.044354 PMC1798412 16396980

[7] U. M. Døhn , B. J. Ejbjerg , M. Court‐Payen , et al., “Are Bone Erosions Detected by Magnetic Resonance Imaging and Ultrasonography True Erosions? A Comparison With Computed Tomography in Rheumatoid Arthritis Metacarpophalangeal Joints,” Arthritis Research & Therapy 8, no. 4 (2006): R110. 10.1186/ar1995 PMC1779369 16848914

[8] T. Aoki , M. Fujii , Y. Yamashita , et al., “Tomosynthesis of the Wrist and Hand in Patients With Rheumatoid Arthritis: Comparison With Radiography and MRI,” AJR. American Journal of Roentgenology 202, no. 2 (2014): 386–390. 10.2214/AJR.12.10029 24450681

[9] U. M. Døhn , B. J. Ejbjerg , M. Hasselquist , et al., “Detection of Bone Erosions in Rheumatoid Arthritis Wrist Joints With Magnetic Resonance Imaging, Computed Tomography and Radiography,” Arthritis Research & Therapy 10, no. 1 (2008): R25. 10.1186/ar2378 PMC2374457 18307764

[10] D. Perry , N. Stewart , N. Benton , et al., “Detection of Erosions in the Rheumatoid Hand: A Comparative Study of Multidetector Computerized Tomography Versus Magnetic Resonance Scanning,” Journal of Rheumatology 32, no. 2 (2005): 256–267. 15693085

[11] C. H. Lee , W. Srikhum , A. J. Burghardt , et al., “Correlation of Structural Abnormalities of the Wrist and Metacarpophalangeal Joints Evaluated by High‐Resolution Peripheral Quantitative Computed Tomography, 3 Tesla Magnetic Resonance Imaging and Conventional Radiographs in Rheumatoid Arthritis,” International Journal of Rheumatic Diseases 18, no. 6 (2015): 628–639. 10.1111/1756-185X.12495 25293500

[12] M. Weiger , K. P. Pruessmann , and F. Hennel , “MRI With Zero Echo Time: Hard Versus Sweep Pulse Excitation,” Magnetic Resonance in Medicine 66, no. 2 (2011): 379–389. 10.1002/mrm.22799 21381099

[13] D. Aletaha , T. Neogi , A. J. Silman , et al., “2010 Rheumatoid Arthritis Classification Criteria: An American College of Rheumatology/European League Against Rheumatism Collaborative Initiative,” Annals of the Rheumatic Diseases 69, no. 9 (2010): 1580–1588. 10.1136/ard.2010.138461 20699241

[14] M. Østergaard , C. G. Peterfy , P. Bird , et al., “The OMERACT Rheumatoid Arthritis Magnetic Resonance Imaging (MRI) Scoring System: Updated Recommendations by the OMERACT MRI in Arthritis Working Group,” Journal of Rheumatology 44, no. 11 (2017): 1706–1712. 10.3899/jrheum.161433 28811353

[15] J. Xu , Y. Hu , R. Zhou , S. Sun , and H. Chen , “Zero Echo Time vs. T1‐Weighted MRI for Assessment of Cortical and Medullary Bone Morphology Abnormalities Using CT as the Reference Standard,” Journal of Magnetic Resonance Imaging 58, no. 3 (2023): 752–760. 10.1002/jmri.28583 36571275

[16] A. Fujisaki , J. Tsukamoto , H. Narimatsu , et al., “Zero Echo Time Magnetic Resonance Imaging; Techniques and Clinical Utility in Musculoskeletal System,” Journal of Magnetic Resonance Imaging 59, no. 1 (2024): 32–42. 10.1002/jmri.28843 37288953

[17] O. J. Sommer , A. Kladosek , V. Weiler , H. Czembirek , M. Boeck , and M. Stiskal , “Rheumatoid Arthritis: A Practical Guide to State‐Of‐The‐Art Imaging, Image Interpretation, and Clinical Implications,” Radiographics 25, no. 2 (2005): 381–398. 10.1148/rg.252045111 15798057

[18] Ü. Aydıngöz , A. E. Yıldız , and F. B. Ergen , “Zero Echo Time Musculoskeletal MRI: Technique, Optimization, Applications, and Pitfalls,” Radiographics 42, no. 5 (2022): 1398–1414. 10.1148/rg.220029 35904982

[19] U. M. Døhn , B. J. Ejbjerg , M. Hasselquist , et al., “Rheumatoid Arthritis Bone Erosion Volumes on CT and MRI: Reliability and Correlations With Erosion Scores on CT, MRI and Radiography,” Annals of the Rheumatic Diseases 66, no. 10 (2007): 1388–1392. 10.1136/ard.2007.072520 PMC1994287 17606464

[20] C. Canella , P. Philippe , V. Pansini , J. Salleron , R.‐M. Flipo , and A. Cotten , “Use of Tomosynthesis for Erosion Evaluation in Rheumatoid Arthritic Hands and Wrists,” Radiology 258, no. 1 (2011): 199–205. 10.1148/radiol.10100791 21045184

[21] M. Østergaard , P. G. Conaghan , P. O'Connor , et al., “Reducing Invasiveness, Duration, and Cost of Magnetic Resonance Imaging in Rheumatoid Arthritis by Omitting Intravenous Contrast Injection—Does It Change the Assessment of Inflammatory and Destructive Joint Changes by the OMERACT RAMRIS?,” Journal of Rheumatology 36, no. 8 (2009): 1806–1810. 10.3899/jrheum.090350 19671817

[22] W. Stomp , A. Krabben , D. Van Der Heijde , et al., “Aiming for a Simpler Early Arthritis MRI Protocol: Can Gd Contrast Administration Be Eliminated?,” European Radiology 25, no. 5 (2015): 1520–1527. 10.1007/s00330-014-3522-1 25636414

